# Zn-Doped Calcium Copper Titanate Synthesized via Rapid Laser Sintering of Sol-Gel Derived Precursors

**DOI:** 10.3390/nano10061163

**Published:** 2020-06-13

**Authors:** Yanwei Huang, Yu Qiao, Yangyang Li, Jiayang He, Heping Zeng

**Affiliations:** 1 State Key Laboratory of Precision Spectroscopy, East China Normal University, Shanghai 200062, China; 5119092722@stu.ecnu.edu.cn (Y.Q.); 51170920010@stu.ecnu.edu.cn (J.H.); 2 Chongqing Institute of East China Normal University, Chongqing 401120, China; 3 School of Materials and Environment, Hangzhou Dianzi University, Hangzhou 310018, Zhejiang, China; liyy@hdu.edu.cn; 4 Jinan Institute of Quantum Technology, Jinan 250101, Shandong, China

**Keywords:** permittivity, impedance, sol-gel, laser sintering

## Abstract

Zn-doped calcium copper titanate (CCTO) was successfully synthesized by rapid laser sintering of sol-gel derived precursors without the conventional long-time heat treatment. The structural, morphological, and crystalline properties were characterized, and the performances of dielectrics and impedance were measured and discussed. The X-ray diffractometer results show that Zn-doped CCTO is polycrystalline in a cubic structure, according to the doping ratio of Ca(Cu_2_Zn)Ti_4_O_12_. Electron microscopy showed that Zn-doped CCTO has a denser microstructure with better uniformness with shrunken interplanar spacing of 2.598 nm for the plane (220). Comparing with undoped CCTO, the permittivity almost remains unchanged in the range of 10^2^–10^6^ Hz, demonstrating good stability on frequency. The electrical mechanism was investigated and is discussed through the impedance spectroscopy analysis. The resistance of grain and grain boundary decreases with rising temperature. Activation energies for the grain boundaries for Zn- doped CCTO were calculated from the slope for the relationship of lnσ versus 1/T and were found to be 0.605 eV, smaller than undoped CCTO. This synthesis route may be an efficient and convenient approach to limit excessive waste of resources.

## 1. Introduction

Today, with the development of the electronics industry, the active demand for better performances, higher capacity and lower cost in various electron devices have become the focus of attention. One promising solution is developing high density capacitors and related materials which possess better dielectric properties, as well as thermal and frequency stability. Calcium copper titanate (CaCu_3_Ti_4_O_12_, CCTO) has been paid much attention due to its novel performances, especially the huge dielectric permittivity (above 10^4^) and wide frequency stability. The value of permittivity is almost frequency-independent up to 10^6^ Hz which shows wide frequency stability. These performances are significant for high density energy storage devices [1,2,3,4,5]. CCTO belongs to the Im3 space group, with a perovskite-like structure with a lattice parameter of 7.391 Å, presenting an extraordinarily high dielectric permittivity ($\varepsilon_{r}$) and moderate dielectric loss (tanδ) [6]. The $\varepsilon_{r}$ is almost independent of frequency in a broad range and it shows excellent phase stability from 20 K to 600 K. This special characteristic could be ascribed to the polarizability mechanism related to the peculiar CCTO crystal structure according to reports [7,8,9]. To date, although different theoretic models have been proposed to explain the electrical properties, consensus has not yet been reached. However, the generally accepted view is the internal barrier layer capacitor (IBLC) model relying on the Maxwell–Wagner polarization [10]. The IBLC model considers that the huge $\varepsilon_{r}$ value could be ascribed to the grains and grain boundaries [2,10,11]. Polycrystalline CCTO usually consists of grains and grain boundaries, which work as semiconductor and insulator, respectively. The grains work as electrodes of connected micro-capacitors and the grain boundaries contribute to the dielectric properties [12]. Researchers have investigated the electrical mechanism of CCTO ceramic using impedance spectroscopy (IS) and demonstrated that it was strongly dependent on the inhomogeneous microstructure, containing conducting grains and insulating boundaries [8]. In addition, comparing with conventional ceramics like PbTiO_3_, BaTiO_3_ or PZT, CCTO demonstrates several disadvantages, such as high dielectric losses at low frequency and low breakdown voltage [13,14]. The disadvantages of CCTO ceramics limit its application in capacitors because higher dielectric loss cannot maintain stored charges for a long time. There are a lot of methods to minimize the dielectric loss and enhance dielectric performance of CCTO materials. Doping of a metallic element at Ca^2+^ or Cu^2+^ sites can depress effectively the oxygen vacancies and alleviate the inner stresses to improve the dielectric properties [15,16,17,18], but the performance of CCTO is much more easily mutable during the synthesis process [4,19].

Various routes to synthesize CCTO have been employed, which include high temperature solid- state sintering, chemical methods, the sol-gel method, co-precipitation, microwave heating, and mechanical mixing methods [15,16,17,18,19,20,21]. The conventional solid-state sintering reaction is a method welcomed by most researchers for preparation of CCTO using powders of oxides or salts as precursors, however it involves higher reaction temperature with long sintering time, even several days. Not only that, it also requires at least two steps to react in the furnace: one is calcination close to 1000 °C and 8–12 h, and the other is sintering at 1150 °C for more than 10 h. Even so, the high temperature solid-state reaction suffers disadvantages of inhomogeneity, and requirement of complex repetitive ball milling with prolonged reaction time at higher temperature. These processes cause excessive consumption of energy which is not suitable for mass production. Other chemical methods, such as the solvothermal method, the sol-gel method, and coprecipitation, allow synthesis of CCTO at lower temperature with a reduced synthesizing time. Although the resultant product is homogeneous in stoichiometric ratio at atomic scale, the final acquisition of pure CCTO is inseparable from the processes of calcination and sintering in the furnace at high temperature [22,23].

Here, we adopted a novel route to synthesize CCTO: rapid laser sintering of sol-gel derived precursors, substituting the conventional furnace. By combining the dual advantages of sol-gel and laser sintering, we first synthesized CCTO precursors by the sol-gel method, which can easily prompt nanostructured and nanosized micro-grain formation. With a focused laser beam through a convex lens directed on the precursors from the sol-gel process, the reactive sintering time is significantly reduced down to several seconds or minutes, while guaranteeing that the CCTO has good crystal structure and high dielectric permittivity. Laser is directed on the precursors to remove the organic ionic group through irradiation at low power first of all and then it induces the solid-state reaction at high power, finally sintered CCTO can be obtained. Meanwhile, the sol-gel route to derive CCTO precursor is beneficial for good stoichiometry, uniform composition, and nanosized particles which can enhance dielectric performance. In this experiment, the precursor for CCTO is first synthesized by the sol-gel process, and then the rapid laser sintering technique is used to substitute the conventional muffle furnace. The obtained Zn-doped CCTO ceramic exhibits high dielectric permittivity and moderate dielectric loss at a wide frequency range, with good frequency-stability. The microstructure, grain and grain boundary resistance were analyzed by studying the impedance spectroscopy, and the conduction mechanism is discussed based on the equivalent circuit constituted of resistors and capacitors for semiconducting grains and insulating boundaries. According to the relationship of lnσ versus 1/T, the activation energies for the grain boundaries were deduced.

## 2. Experimental Details

CCTO and Zn-doped CCTO precursors were obtained first by the sol-gel method. The original materials included Ti(OC_4_H_9_)_4_(99%), Ca(CH_3_COO)_2_·H_2_O(99%), Cu(NO_3_)_2_·3H_2_O(99%), and Zn(CH_3_COO)_2_·2H_2_O(99%), which are bought from the Sinopharm Chemical Reagent Co., Ltd. (SCR, China). Glacial acetic acid (bought from SCR) was used as a complexing agent. Ti^4+^, Cu^2+^, Ca^2+,^ and Zn^2+^-containing sol were, respectively, made from the raw materials according to the molar ratio 4:3:1 and 4:2:1:1. A weighed amount of Ti(OC_4_H_9_)_4_ was added in ethanol by stirring and with glacial acetic acid through magnetic stirring formed solution A. A clear and blue solution was formed by mixing and stirring a mixture of Cu(NO_3_)_2_·3H_2_O with ethanol and then added into solution A to form solution B through vigorous stirring with a magnetic stirrer. Similarly, an appropriate amount of Ca(CH_3_COO)_2_·H_2_O with solvent deionized water was stirred to form solution C by stirring and then was mixed with solution B. Zn^2+^-containing sol was prepared similarly from Zn(CH_3_COO)_2_·2H_2_O with deionized water. Finally, a blue-green sol was obtained after stirring for 1h and aging for 12 h. The sol was then baked at 80 °C for 12 h to form a dry gel and then it was ground into fine powder. After drying at 150 °C for 8 h, the fine powders were again ground in an agate mortar for 1 h ready for laser treatment.

A multimode diode laser (Optotools, 980 nm, CW, 1200 W, DILAS, Mainz-Galileo-Galilei, Germany) was well adjusted and then directed on samples through a quartz convex lens. In order to make the precursors react uniformly with laser radiation, we adjusted the laser in defocus so that the spot could cover the samples. The fine dry gel powders were thermally treated by laser irradiation at a relatively low power to remove the organic matter and the CCTO or Zn-doped CCTO precursor powders were thus obtained. As-prepared precursors were pressed in an oil press into pellets with thickness of 2 mm, and diameter of 10 mm under a pressure of 100 MPa. The pellets were put into a copper crucible for laser sintering and the solid- state reaction occurred for the precursors in the pellet when irradiated under high laser power. To guarantee the structural homogeneity of samples, we adjusted the laser spot diameter in the defocusing state to cover the pressed pellet. Then the pellet was cooled down to room temperature naturally and the finally obtained product was CCTO-based ceramic.

X-ray diffraction (XRD) patterns for samples were collected on a Rigaku Ultima VI X-ray diffractometer (Cu-Kα radiation, λ = 1.5418Å, Rigaku, Tokyo, Japan). The morphologies and microstructures of CCTO were measured by scanning electron microscope (SEM, ZEISS MERLIN Compact, Oberkochen, Baden-Württemberg, Germany) and high resolution transmission electron microscopy (HRTEM, FEI Tecnai G20, Hillsboro, OR, USA). Raman experiments were performed using a Renishaw in Via Raman spectrometer (London, UK) with 532 nm excitation. For dielectric measurements, electrodes of silver paste were painted on both sides of the thin ceramic cylindrical piece. The dielectric constants, dielectric loss, and complex impedance of CCTO-based ceramic samples were measured with a high-precision LCR meter (HP 4990A; Agilent, Palo Alto, CA, USA).

## 3. Results and Discussion

**Figure 1:**
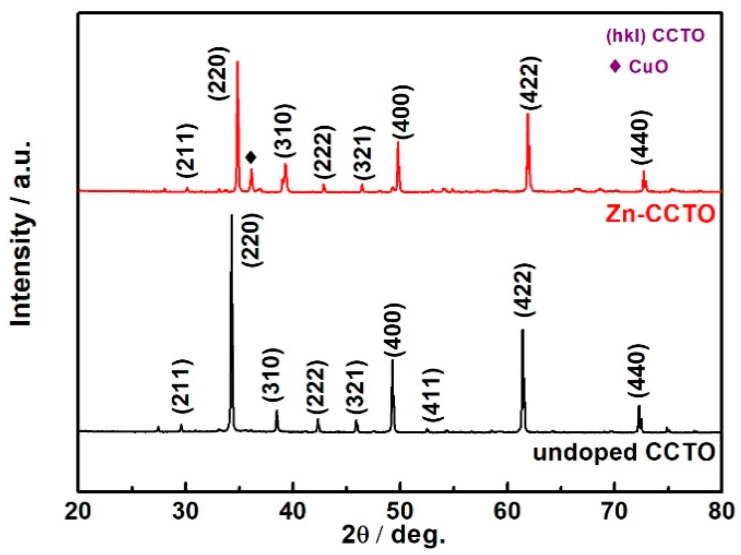
X-ray diffraction (XRD) patterns of undoped and Zn-doped calcium copper titanate (CCTO) prepared by rapid laser sintering of sol-gel derived precursors.

**Figure 2:**
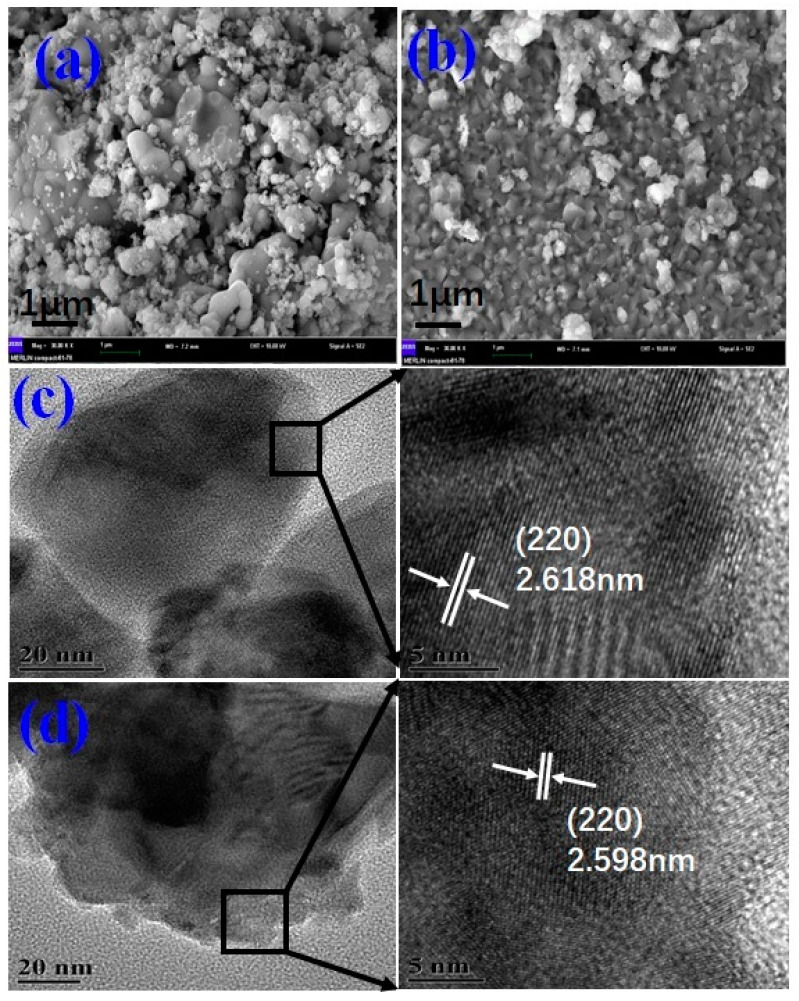
The SEM and HRTEM images for prepared undoped (**a**,**c**) and Zn-doped CCTO (**b**,**d**).

The precursors were thermally treated by laser irradiation at 30 W for 10 min to remove the organic group. The precursor pellets were sintered under laser at 120 W for 5 s to form the CCTO- based ceramics. Figure 1 shows XRD spectra of the prepared ceramics. The diffraction peaks identified were found to be consistent with the diffraction planes of body-centered cubic structure of CCTO, agreeing with the PDF file No. 75-1149. The major peaks include (211), (220), (310), (222), (321), (400), (411), (422), and (440) for CCTO and Zn-doped CCTO samples. We can see that the samples demonstrate better crystalline quality. The Zn-doped CCTO also has a pseudo-cubic structure according to the doping ratio of Ca(Cu_2_Zn)Ti_4_O_12_. The main diffraction peaks almost show no splitting peak and only a small peak appears at 36.1 which can be attributed to peak (101) of CuO. Meanwhile, the Zn-doped CCTO shows that the diffraction peak moved to a high diffraction angle, signifying d-spacing reduction which could easily result in smaller grain size to enhance the compactness of the ceramics. Figure 2 shows SEM and HRTEM images of prepared CCTO and Zn- doped CCTO. It shows that the doped CCTO has better uniformity in particle size and better densification. Figure 2c,d present the HRTEM images which clearly demonstrate a well-defined crystalline lattice structure. The determined interplanar spacing of the plane (220) shows it was shrunken slightly from 2.618 nm to 2.598 nm with Zn-doping which is consistent with analysis of the results of XRD and SEM.

**Figure 3:**
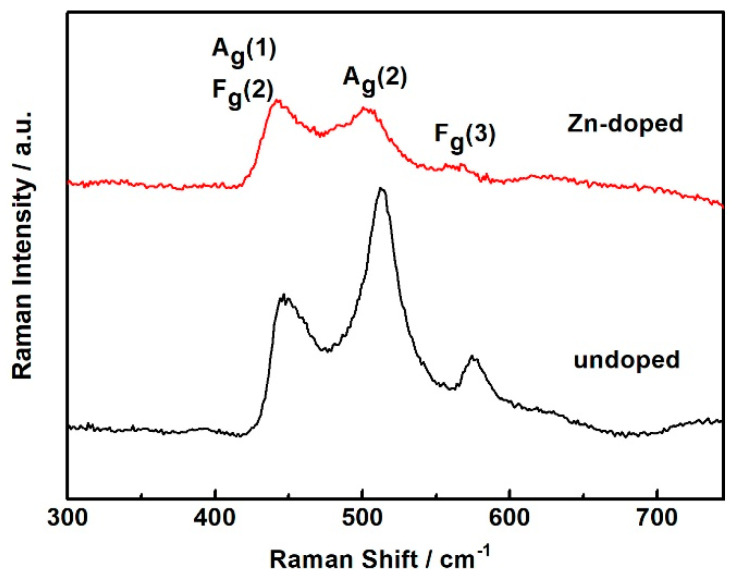
Raman measurement for undoped and Zn-doped CCTO prepared by laser treatment of sol- gel derived precursors.

Raman spectra measurements were performed on samples after laser sintering of the precursors, shown in Figure 3. As the modes of Raman show, there are peaks located at 446 cm^−1^, 513 cm^−1^, and 576 cm^−1^ for undoped CCTO which is in accord with previous reports [24,25,26]. The Raman shift of 446 cm^−1^ corresponds to the modes of A_g_(1) and F_g_(2), and the peaks at 513 and 576 cm^−1^ correspond to A_g_(2) and F_g_(3), respectively. The modes of A_g_(1), A_g_(2) and F_g_(2) are ascribed to lattice vibrations in TiO_6_, and F_g_(3) reflects the vibration mode of adverse stretching for the O–Ti–O bond [24,27]. The Raman vibration modes of Zn-doped CCTO show a shift to lower frequencies at 442 cm^−1^, 506 cm^−1,^ and 570 cm^−1^, assigned to A_g_(1)/F_g_(2), A_g_(2), and F_g_(3), respectively. This could be attributed to Zn ion incorporation affecting the structure of CCTO. The weakness of the relative intensity for the Raman peaks in Zn-doped CCTO, especially for the A_g_(2) mode, demonstrates that Zn doping changes slightly the lattice vibrations of the titanium–oxygen octahedron.

**Figure 4:**
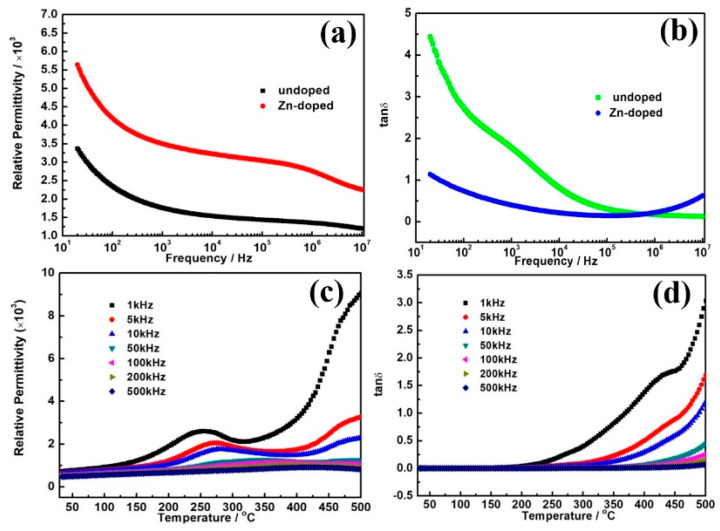
The relationships of frequency and permittivity, loss for undoped (**a**) and Zn-doped CCTO (**b**). Permittivity and loss for Zn-doped CCTO measured from 30 to 500 °C (**c**,**d**).

Figure 4a,b shows the frequency relationship of permittivity and tan δ for undoped and Zn- doped CCTO in the range of 10–10^7^ Hz. The samples show permittivity of above 10^3^ at the broad frequency region, and the dielectric permittivity decreases gently over the entire frequency range shown in Figure 4a. It shows that the permittivity almost remains unchanged in the range of 10^2^–10^6^ Hz, demonstrating good stability on frequency. The frequency-stability of the permittivity should be closely associated with size of grain, density of grain boundaries, and the compactness of ceramics. The Zn-doped CCTO has higher permittivity than undoped CCTO. This could be ascribed to the denser structure and finer particles which can be seen from the SEM images and HRTEM. Above 10^6^ Hz, the permittivity descends linearly, which could be attributed to Debye-like relaxation [28,29]. In Figure 4b we can see that tan δ of the specimen declines first in low-frequency to a platform in middle-frequency and then gently increases in high-frequency. The Zn-doped CCTO demonstrates lower dielectric loss than the undoped sample over the entire frequency region, especially in the low frequency range. Therefore, Zn doping effectively reduces the dielectric loss and enhances the dielectric properties. The mechanism of giant dielectric permittivity of CCTO is still a hotspot of controversy, but people generally believe that the behavior of CCTO could be related to the microstructure which may play a vital role for the huge permittivity. Several techniques have been adopted to improve the dielectric properties of CCTO, including nanosizing, recombination, modifying etc. Among them element doping in based materials is a simple and efficient method. A lot of elements have been doped into CCTO, but unanticipated results often occur, with accompanying dielectric loss. Most literatures reported element doping raised the permittivity as well as loss arising [9,18,30,31]. Said Senda et al. [32] reported Ni-doped CCTO prepared by the routine solid state reaction method. They studied on grain growth, morphological evolution with Ni doping content and found the dielectric properties including the permittivity, loss, and resistivity were improved. It was interpreted that dopants could easily segregate between the grain boundaries, and further depress the grain growth which changes the Cu/Ti stoichiometry. Finally, it greatly influences the microstructure of materials. This is in agreement with results of this work. Sonia De Almeida-Didry et al. [33] studied alumina doped CCTO prepared by the core-shell approach and they found that the grain boundaries of CCTO materials play a key role in the huge permittivity by controlling the density of grain boundaries. They demonstrated that core-shell design is an efficient technique to obtain a high dielectric constant, which shows obvious performance improvement. Furthermore, the various synthesized methods for doped CCTO have been very important to improve the dielectric performance. M.A. dela Rubia et al. [34] reported the dielectric properties for Hf doping CCTO ceramics prepared by comparing these two synthesizing routes. They concluded that the reactive sintering method is better than the conventional synthesis technique. The reactive sintering method offers the convenience for Hf incorporation in the CCTO lattice. Dong Xu et al. [35] reported CCTO with Zn element doping by the sol-gel method and found Zn-doping does not improve the dielectric properties because of the mesoporous structure of the synthesized materials. Therefore, the improved sol-gel method here with laser treatment can effectively enhance the dielectric properties. It can be concluded that a compact structure composed of a number of smaller grains can benefit the improvement of overall dielectric performance.

Figure 4c,d shows the permittivity and loss of Zn-doped CCTO with temperature at fixed frequencies. The values of dielectric permittivity obviously increase with rising temperature and decrease with incremental frequency. At higher temperature region (>400 °C), the dielectric permittivity increases with a large amplitude, which is attributed to enhanced conductivity by high temperature. The decrease in dielectric permittivity at higher frequencies could be explained in respect of the space charge polarization occurring at the interface [36], which is due to heterogeneous microstructures. This polarization mechanism is enhanced at higher temperature which could be ascribed to the direct current conductivity rising. Thus, the polarization arises quickly in permittivity at high temperature and low frequencies. Figure 4d shows the changes in the dielectric loss (tan δ) of the Zn-doped CCTO ceramic with temperature at some fixed frequencies. It can be seen that the tan δ demonstrates a low platform from 30 to 250 °C, then an obvious increase above the temperature of 250 °C for all given frequencies. However, tan δ remains at lower values at higher frequency. The dielectric loss of Zn-doped CCTO materials is strongly temperature dependent.

**Figure 5:**
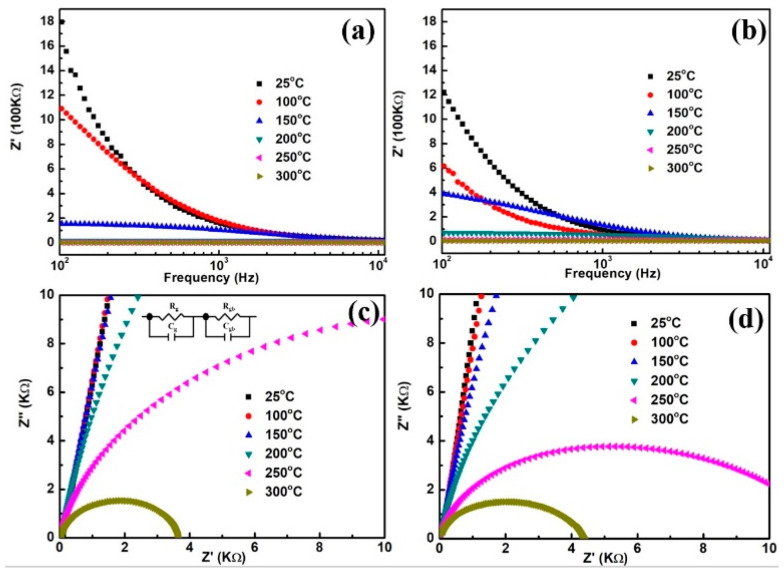
The real part (Z’) versus frequency (**a**,**b**) and complex impedance plots (**c**,**d**) for undoped CCTO (**a**,**c**) and Zn-doped CCTO (**c**,**d**), respectively, with a measured temperature of 25–300 °C. Inset in (**c**) shows the two parallel RC equivalent circuits in series.

Figure 5a,b shows that the real part of the impedance changes at some fixed temperatures with frequency for undoped and Zn-doped CCTO, respectively. Below 10^3^ Hz when the temperature increases, the values of Z’ decreases and then the Z’ values almost merge above 10^4^ Hz. The different Z’ values indicate the various electrical properties related to the microstructures. For the undoped CCTO, the Z’ values are more divergent than the Zn-doped sample. Researchers attributed this dispersion at lower frequency to reduction of space charge polarization and discharging, which may be attributed to charge carrier hopping between para-electric phases [37,38,39]. Figure 5c,d shows the Nyquist plot for undoped and Zn-doped CCTO with a measured temperature of 25–300 °C. Impedance spectroscopy is an efficient tool to figure out the contribution mechanism of grains, boundaries, and interfaces to the total electrical conduction performance of materials by the equivalent RC circuit model. The curves commonly consist of two semicircular arcs standing for the resistances of the grain (R_g_) and the grain boundary (R_gb_).

Respectively, it can be seen that the data in this work only show arcs partly due to the measuring range limit. According to the approximate conduction mechanisms of the IBLC model, the samples are formed by conducting grains and insulating boundaries. The inset of Figure 5c shows the RC equivalent circuit. It is apparent from Figure 5c,d that the resistance values of the grain and grain boundaries decrease with rising temperature, which is consistent with the results of enhanced conductivity with high temperature from Figure 4c,d. The Zn-doping affected the resistances of the CCTO bulk (R_g_) and boundary (R_gb_) which can be seen as the specimen demonstrates a smaller diameter of the semicircle than the undoped one around 200 °C. Moreover, the decreases in diameter of the semicircle with rising temperature indicated that the resistance of the samples demonstrates a negative temperature coefficient [40]. Based on the equivalent circuit, the fitted values with Z- View software, are shown in Table 1.

**Figure 6:**
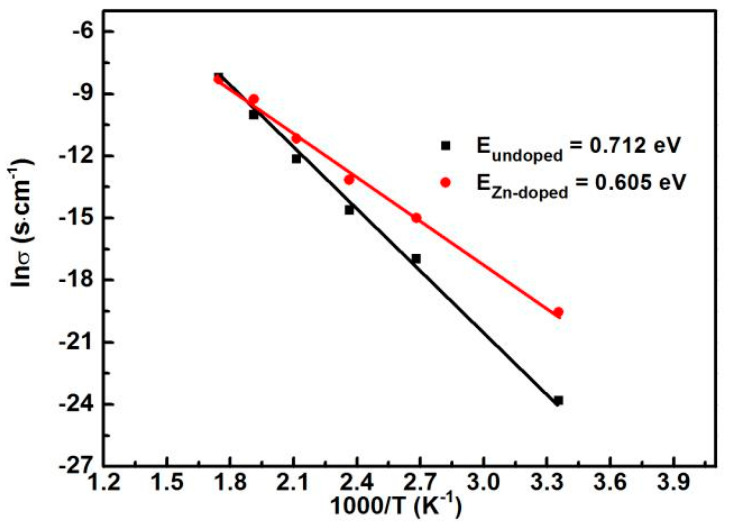
lnσ vs. temperature for undoped and Zn doping CCTO in the form of Arrhenius plots.

To reveal the effectiveness of grain boundaries on Zn-doped CCTO, we studied the dependence of temperature on the conductivity of grain boundaries, and the grain boundary resistance (R_gb_) was deduced from the impedance spectra as described in Table 1. According to the Arrhenius equation, the resistance has a close relationship with temperature, which could be summarized in this equation:

(1) $$\sigma \left(T\right)=\sigma \exp\left(-\frac{E}{\mathrm{KT}}\right)$$

where σ the pre-exponential factor, E presents the activation energy of the grain boundary, K is the Boltzmann constant and T is the absolute temperature, respectively. For the plot of lnσ vs. 1/T as shown in Figure 6, the solid lines show the results fitted using the Arrhenius equation. The experimental data approximately obeys the equation. The calculated activation energy for grain boundaries E_gb_ is 0.712 eV and 0.605 eV for undoped and doped CCTO, respectively, which is close to the reported values. The decrease in activation energy of E for the Zn-doped sample suggests a change in electrical conductivity of the grain boundaries, which is due to the intensive motion of the charge carriers accumulated at the boundaries.

## 4. Conclusions

Zn-doped CCTO was prepared using a novel method of rapid laser sintering of sol-gel derived precursors. By comparing with undoped CCTO, the microstructure, dielectric properties, and impedance response were investigated in detail. HRTEM images demonstrate a well-defined crystalline lattice structure. The determined interplanar spacing of the plane (220) shows that it was shrunken slightly from 2.618 nm to 2.598 nm with Zn-doping. Raman spectra analysis demonstrated that the Zn doping changes slightly the lattice vibrations of the titanium–oxygen octahedron. The permittivity almost remains unchanged in the range of 10^2^–10^6^ Hz, demonstrating good stability on frequency. The impedance mechanisms were discussed according to the IBLC model by impedance spectroscopy analysis, and the results show that Zn-doping obviously affected the grain and grain boundary resistance. The activation energy for the grain boundaries was deduced from the slope of lnσ versus 1/T. The activation energy of $E_{A}$ for the Zn-doped sample is 0.605 eV, smaller than the undoped sample, which suggests increased charge carrier motion at the grain boundaries. This technique overcomes the shortcomings of long-time thermal energy supply by a furnace and presents references to synthesize the ceramic materials through a combination of soft-chemical methods.

## Figures and Tables

**Table 1:** Grain and grain boundary resistance for undoped and Zn-doped calcium copper titanate (CCTO) at temperature range of 25–300 °C.

Temperature (°C)	Undoped		Zn-Doped	
	R_g_ (Ω)	R_gb_ (Ω)	R_g_ (Ω)	R_gb_ (Ω)
25	906.7	8,998,000,000	837.4	312,700,000
100	422.7	23,223,000	406.9	3,288,100
150	223.8	2,225,400	276.2	512,790
200	105.9	188,050	102.2	70,520
250	90.5	22,282	50.6	10,454
300	50.0	3660	40.2	4076
